# Accumulation of sublethal radiation damage and its effect on cell survival

**DOI:** 10.1088/1361-6560/aca5e7

**Published:** 2022-12-19

**Authors:** Oleg N Vassiliev

**Affiliations:** 1 Department of Radiation Physics, The University of Texas, MD Anderson Cancer Center, Houston, TX 77030, United States of America

**Keywords:** cell survival modelling, RBE calculation, proton therapy, hadron therapy

## Abstract

*Objective.* Determine the extent of sublethal radiation damage (SRD) in a cell population that received a given dose of radiation and the impact of this damage on cell survival. *Approach.* We developed a novel formalism to account for accumulation of SRD with increasing dose. It is based on a very general formula for cell survival that correctly predicts the basic properties of cell survival curves, such as the transition from the linear-quadratic to a linear dependence at high doses. Using this formalism we analyzed extensive experimental data for photons, protons and heavy ions to evaluate model parameters, quantify the extent of SRD and its impact on cell survival. *Main results.* Significant accumulation of SRD begins at doses below 1 Gy. As dose increases, so does the number of damaged cells and the amount of SRD in individual cells. SRD buildup in a cell increases the likelihood of complex irrepairable damage. For this reason, during a dose fraction delivery, each dose increment makes cells more radiosensitive. This gradual radosensitization is evidenced by the increasing slope of survival curves observed experimentally. It continues until the fraction is delivered, unless radiosensitivity reaches its maximum first. The maximum radiosensitivity is achieved when SRD accumulated in most cells is the maximum damage they can repair. After this maximum is reached, the slope of a survival curve, logarithm of survival versus dose, becomes constant, dose independent. The survival curve becomes a straight line, as experimental data at high doses show. These processes are random. They cause large cell-to-cell variability in the extent of damage and radiosensitivity of individual cells. *Significance.* SRD is in effect a radiosensitizer and its accumulation is a significant factor affecting cell survival, especially at high doses. We developed a novel formalism to study this phenomena and reported pertinent data for several particle types.

## 1. Introduction

Substantial data have been accumulated on discrepancies between the linear-quadratic (LQ) model of cell survival and experimental data (Elkind and Sutton 1959, Kirkpatrick *et al* 2008, Iwata *et al* 2009). The data suggest that the slope of a survival curve, $\mathrm{ln}S$ versus dose, tends to a finite limit as the dose increases. In other words, the curve attains a constant slope and $\mathrm{ln}S$ becomes a linear function of dose. One of the earliest radiobiological models that accounted for this property was a model (Scholz and Kraft 1994) that is a precursor of the local effect model (Kramer and Scholz 2000). Multiple models were proposed to improve agreement with experimental data and offering various paths to achieving a constant slope. McKenna and Ahmad (2009) reviewed the most prominent models. Several models (Scholz and Kraft 1994, Guerrero and Li 2004, Park *et al* 2008) were modifications of the LQ formula. Other standard models were also explored in this context, such as the multi-target (McKenna and Ahmad 2009) and multi-hit models (Vassiliev 2012, Zhao *et al* 2020). Models based on a realistic mechanism included the lethal-potentially lethal model (Curtis 1986) and the compartmental form of the linear-quadratic-linear model (Carlone *et al* 2005, Guerrero and Carlone 2010). However, the actual processes are rather complex, and there is still a potential for advancing mechanism-based models. Furthermore, these studies were largely focused on x-rays. The current expansion of hadron therapy has generated strong interest in accurate models of cell survival for hadrons and motivated new research in this area.

The current study is based on a very general formula for cell survival applicable to x-rays and hadrons. This formula is consistent with the LQ model at low doses and produces a constant slope of cell survival curves at high doses. This formula proved to be a useful tool for analysis of sublethal radiation damage (SRD) caused by different particles, its accumulation and impact on cell survival. At the same time, our methodology is minimalistic in terms of the mathematics involved. The key finding of the present study is that SRD makes affected cells more sensitive to radiation. SRD impacts cells at random and, as it accumulates with increasing doses, a cell population becomes heterogeneous in terms of radiosensitivity. Above a certain dose level, the most radiosensitive subpopulation becomes the largest. This results in the survival curve attaining a constant slope, which is a characteristic of cells with inhibited repair.

## 2. Methods and materials

### 2.1. A general cell survival formula

In our model formulation, we used the following terminology:

• *Track*: A track comprises all inelastic interactions of a single primary particle produced by the source, and by all secondary particles, such as delta electrons and nuclear recoils.
• *Sensitive volume (SV)*: SV is a subcellular structure sensitive to radiation, the target volume.
• *Energy deposition event (‘hit’)*: In each event a particle undergoes at least one inelastic interaction within the SV and deposits energy. If two or more particles that belong to the same track, such as those produced by the same parent particle, enter the SV and deposit energy, this is counted as one event. With this convention, use of the Poisson distribution for the number of hits is fully justified. The Poisson distribution was introduced to describe the statistics of energy deposition events in previous studies (for exanple, Kellerer and Rossi 1972, Rossi and Zaider 1996). Since then it has been an integral part of the microdosimetric formalism.

The LQ model accounts for intratrack damage and for that caused by intertrack interactions involving two tracks. We use a more general formula that accounts for interactions between any number of tracks and offers a method for calculating the respective probabilities. The formula is based on the total probability equation for the probability *S* of a cell surviving a given dose of radiation:

\begin{eqnarray*}S={h}_{0}{s}_{0}+{h}_{1}{s}_{1}+{h}_{2}{s}_{2}+{h}_{3}{s}_{3}+\ldots ,\end{eqnarray*}

where *h*_*k*_ is the probability of the SV receiving *k* hits and *s*_*k*_ is the probability of a cell surviving *k* hits. These probabilities depend on several physical and biological factors including, for example, linear energy transfer (LET), dose rate and damage repair rate. Analysis of these factors is beyond the scope of the current study. Instead, we have focused on general properties of cell survival curves at high doses where deviations from the LQ model had previously been reported.

The number of hits *k* is Poisson distributed:

\begin{eqnarray*}{h}_{k}=\displaystyle \frac{{N}^{k}}{k!}\exp (-N),\end{eqnarray*}

where *N* is the average number of hits at a dose *D*. For *N*, we use an expression from microdosimetry (Rossi and Zaider 1996, Vassiliev 2017):

\begin{eqnarray*}N=\displaystyle \frac{D}{{z}_{F}}.\end{eqnarray*}

Parameter *z*_*F*_ (Gy) is the frequency average specific energy. It is defined as the average energy deposited in a volume, per unit mass, per event. It strongly depends on SV size, which will help us estimate SV size. This leads to our main formula for cell survival:

\begin{eqnarray*}S=\left[1+{{Ns}}_{1}+\displaystyle \frac{{N}^{2}}{2}{s}_{2}+\displaystyle \frac{{N}^{3}}{3!}{s}_{3}+\ldots \right]{\mathrm{\exp }}(-N)={\mathrm{\exp }}(-D{/z}_{F})\displaystyle \sum _{i=0}^{\infty }\displaystyle \frac{{s}_{i}{\left(D{/z}_{F}\right)}^{i}}{i!}.\end{eqnarray*}

Our model, equation (4), is based on a minimal number of assumptions and predicts the basic properties of cell survival curves, as described below.

(1) *Cell survival is LQ at low doses*. To prove this, we derive a power series expansion of $\mathrm{ln}S$ versus dose. Terminated after the quadratic term, this series has the same dose dependence as does the LQ model: $-\mathrm{ln}S=\alpha D+\beta {D}^{2}$. From this expansion, we find the relationship between *α* and *β* of the LQ model with parameters of our model:
  \begin{eqnarray*}\alpha =-\mathop{\mathrm{lim}}\limits_{D\to 0}\displaystyle \frac{\partial }{\partial D}\mathrm{ln}S.\end{eqnarray*}
  \begin{eqnarray*}\displaystyle \frac{\partial }{\partial D}\mathrm{ln}S=-\displaystyle \frac{1}{{z}_{F}}+\left[\displaystyle \sum _{i=1}^{\infty }\displaystyle \frac{{{is}}_{i}{\left(D{/z}_{F}\right)}^{i-1}}{i!{z}_{F}}\right]/\left[\displaystyle \sum _{i=0}^{\infty }\displaystyle \frac{{s}_{i}{\left(D{/z}_{F}\right)}^{i}}{i!}\right].\end{eqnarray*}
  In the *D* → 0 limit, in each of the two sums only one term is nonzero: *i* = 1 in the first sum and *i* = 0—in the second. Then, the result is
  \begin{eqnarray*}\alpha =\displaystyle \frac{1-{s}_{1}}{{z}_{F}}.\end{eqnarray*}
  Calculations for *β* are very similar:
  \begin{eqnarray*}\beta =-\displaystyle \frac{1}{2}\mathop{\mathrm{lim}}\limits_{D\to 0}\displaystyle \frac{{\partial }^{2}}{\partial {D}^{2}}\mathrm{ln}S.\end{eqnarray*}
  \begin{eqnarray*}\begin{array}{rcl}\displaystyle \frac{{\partial }^{2}}{\partial {D}^{2}}\mathrm{ln}S &amp; = &amp; \left[\displaystyle \sum _{i=2}^{\infty }\displaystyle \frac{i(i-1){s}_{i}{\left(D{/z}_{F}\right)}^{i-2}}{i!{z}_{F}^{2}}\right]/\left[\displaystyle \sum _{i=0}^{\infty }\displaystyle \frac{{s}_{i}{\left(D{/z}_{F}\right)}^{i}}{i!}\right]\\ &amp; &amp; -{\left[\displaystyle \sum _{i=1}^{\infty }\displaystyle \frac{{{is}}_{i}{\left(D{/z}_{F}\right)}^{i-1}}{i!{z}_{F}}\right]}^{2}/{\left[\displaystyle \sum _{i=0}^{\infty }\displaystyle \frac{{s}_{i}{\left(D{/z}_{F}\right)}^{i}}{i!}\right]}^{2}.\end{array}\end{eqnarray*}
  \begin{eqnarray*}\beta =\displaystyle \frac{{s}_{1}^{2}-{s}_{2}}{2{z}_{F}^{2}}.\end{eqnarray*}
  In equation (10), *s*_2_ is the probability of surviving the first and the second hits. According to the definition of conditional probability (Ash 2008), *s*_2_ is the product of *s*_1_ and conditional probability *s*(2∣1) of surviving the second hit after receiving the first hit. The probability of surviving the second hit, *s*(2∣1), cannot be higher than *s*_1_. This means that *β* is nonnegative. We use equations (7) and (10) to link our cell survival model with experimental survival data.
(2) *Survival curves have a constant slope in four situations*.
  • Inhibited repair: *s*_*i*_ = 0 for all *i* > 0.
  • High-LET particles: a single hit inflicts irreparable damage, *s*_*i*_ = 0 for all *i* > 0.
  • Accumulated damage at high doses: this situation is the focus of our study. As the dose increases, damage accumulates and at a certain level becomes irreparable. Then, a number *k* exists such that *s*_*i*_ = 0 for all *i* > *k*. In this case the infinite sum in equation (4) becomes a polynomial of order *k* in dose, and the high-dose slope, *α*_∞_, can be calculated exactly. To find it, we calculate the first derivative of $\mathrm{ln}S$ using equation (4), and take limit *D* → ∞ . The result is a dose-independent constant:
    \begin{eqnarray*}{\alpha }_{\infty }=-\mathop{\mathrm{lim}}\limits_{D\to \infty }\displaystyle \frac{\partial }{\partial D}\mathrm{ln}S=\displaystyle \frac{1}{{z}_{F}}.\end{eqnarray*}
    This means that a survival curve, $\mathrm{ln}S$ versus dose, becomes a straight line as dose increases:
    \begin{eqnarray*}S\to \exp \left(-\displaystyle \frac{D}{{z}_{F}}\right).\end{eqnarray*}
  • Independent repair (IR): repair of damage from a hit is independent of damage from other hits. This may happen when each hit causes limited and localized damage, and the damaged sites are well separated one from another. In this case ${s}_{i}={s}_{1}^{i}$, and the infinite sum in the square brackets in equation (4) can be calculated, resulting in:
    \begin{eqnarray*}{S}_{{IR}}=\exp \left[-\displaystyle \frac{D}{{z}_{F}}(1-{s}_{1})\right].\end{eqnarray*}
    This mechanism may explain, at least partially, the properties of cell survival data for ultrasoft x-rays. For example, carbon K-shell x-rays have energy 0.278 keV and produce electrons with a range of 7 nm in water (Goodhead *et al* 1979). They deposit ‘highly localized and very low energy’ (Goodhead *et al* 1979). These x-rays have a higher biological effectiveness than 20 keV *μ*m^−1^ helium ions, and produce cell survival curves with zero (Goodhead *et al* 1979) or small curvatures: *α*/*β* = 8.5 Gy (Thacker *et al* 1986) and 10.1 Gy (Frankenberg *et al* 1995). Then, there is a contradiction: energy deposited by multiple electron tracks is needed to cause lethal damage, but the survival curve is almost a straight line typical for predominantly intratrack (single-track) damage. The IR mechanism resolves this contradiction.
(3) *Cell survival curves have a shoulder that disappears with increasing LET*. Comparing equations (7) and (11), we see that the initial slope *α* is smaller than the high-dose slope *α*_∞_. This indicates a curved shape resembling a shoulder. The difference between the two slopes is given by *s*_1_. As LET increases, *s*_1_ decreases, because damage from a single hit increases, which results in the shoulder disappearing.

### 2.2. Calculation of *z*_*F*_ from cell survival data

We use two methods to derive *z*_*F*_ from experimental cell survival data. In method 1 we find upper bounds, ${z}_{F,\max }$. This method requires only experimental *α* and *β*. A disadvantage is that it produces the upper bound but not the actual *z*_*F*_ for a given curve. Method 2 overcomes this problem. It is based on asymptotic properties of cell survival at high doses and/or high LET values. In both cases, the slope of a survival curve is ${z}_{F}^{-1}$. The high-dose asymptote is given by equation (12), and the high-LET asymptote was derived previously (Vassiliev *et al* 2017). The latter result also follows from equation (4), because *s*_*i*_ → 0 for *i* > 0 when LET → ∞ . Hence, *z*_*F*_ can be determined from a linear regression that is limited to the dose or LET range where this asymptotic behavior is achieved. Method 2 requires cell survival data that extend to either low cell survival levels or high LET values.

*Method 1*. First, we find the bounds for *α* and *β*. In equation (7), *s*_1_ is a probability, 0 ≤ *s*_1_ ≤ 1, which gives us

\begin{eqnarray*}0\leqslant \alpha \leqslant \displaystyle \frac{1}{{z}_{F}}.\end{eqnarray*}

In equation (10), *s*_2_ is also a probability but its upper bound is ${s}_{1}^{2}$, as we discussed above. Then,

\begin{eqnarray*}0\leqslant \beta \leqslant \displaystyle \frac{{\left(1-\alpha {z}_{F}\right)}^{2}}{2{z}_{F}^{2}}.\end{eqnarray*}

From equations (14) and (15) we find the bounds for *z*_*F*_:

\begin{eqnarray*}0\leqslant {z}_{F}\leqslant \displaystyle \frac{1}{\alpha +\sqrt{2\beta }}\equiv {z}_{F,\max }.\end{eqnarray*}

In method 1, using equation (16) and measured *α* and *β* values, we find ${z}_{F,\max }$. Then, using the results for ${z}_{F,\max }$, we calculate lower bounds for SV size.

*Method 2*. To find *z*_*F*_, we have shown that in the high dose and/or high LET limits, the slope of a survival curve is 1/*z*_*F*_. In both cases, the mechanism is the same: one additional hit kills the cell. For high-LET particles this occurs because one hit delivers a large amount of energy to the SV. In the case of low-LET particles at high doses, the cell has already accumulated such extensive SRD that receiving any additional amount of energy is lethal. These arguments are supported by the high-dose asymptotic formula for the probability of cell survival (equation (12)), which coincides with the probability of zero hits (equations (2), (3)). Hence, we find *z*_*F*_ from the slope of measured cell survival curves at high doses and/or high LET. We chose for these calculations two studies: Elkind and Sutton (1959) and Miller *et al* (1995). Elkind and Sutton reported cell survival of V79 cells irradiated by 55 kV x-rays, at survival levels extending below 0.001. Miller *et al* reported survival of C3H 10T1/2 cells for several particles with LET values from 3.8 to 600 keV *μ*m^−1^.

### 2.3. Calculation of SV size

SV size is an important parameter for modelling cell survival. Also, calculation of SV size is a test for the approach we develop. Assuming that SV is a sphere, we find its radius, *R*, by solving this equation (Kase *et al* 2006, Vassiliev *et al* 2017) for a given *z*_*F*_:

\begin{eqnarray*}{z}_{F}=\displaystyle \frac{{y}_{F}(E,R)}{\pi \rho {R}^{2}}.\end{eqnarray*}

Here, *E* is the particle energy, *ρ* is the density of SV material, 1 g cm^−3^, and *y*_*F*_ is the frequency-average lineal energy (Rossi and Zaider 1996), which depends weakly on *R*. For this reason the right hand side of equation (17) is a monotonically decreasing function of *R* and, therefore, a unique solution of equation (17) exists. To verify this property for a specific radiation type, measured and caclulated with Monte Carlo *y*_*F*_ values available in the literature can be used. Examples of such data are given in section 3.2. For calculation of SV size we used ${z}_{F,\max }$, which produced the lower bound for the SV size, *R*_min_.

## 3. Results and discussion

### 3.1. Calculation of *z*_*F*_ from cell survival data

#### 3.1.1. Method 1

**Figure 1.**
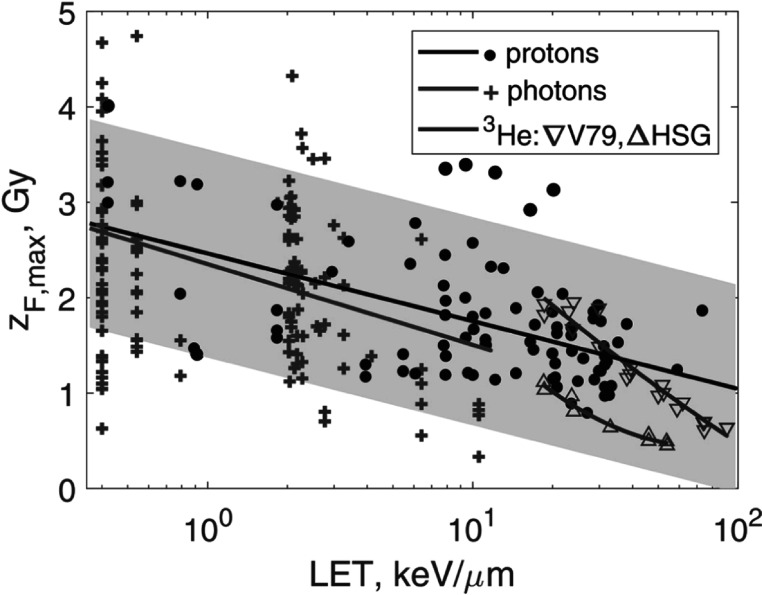
Upper bounds for *z*_*F*_ derived by method 1 (equation (16)) from experimental *α* and *β* values. Black circles indicate data for protons for multiple cell lines; red crosses, photons for multiple cell lines; triangles, ^3^He ions (down/up triangles V79/HSG cells). Solid lines show linear and polynomial regressions, and the shaded area is the 95% confidence interval for the proton regression. (*α*, *β*) were taken from Paganetti (2014), Vassiliev *et al* (2020), and Furusawa *et al* (2000) for protons, photons, and ^3^He ions, respectively.

*Protons*. From a review on proton relative biological effectiveness (Paganetti 2014) we selected cell survival data from experiments with monoenergetic protons. Our dataset comprised 91 (*α*, *β*) pairs for protons with LET values from 0.42 to 73.2 keV *μ*m^−1^. For each experiment, we calculated ${z}_{F,\max }$ according to equation (16) and fit these data with a linear regression ${z}_{F,\max }=a\cdot \mathrm{ln}{\mathrm{LET}}+b$ using the Huber robust method (Huber 1981). The regression coefficients were: *a* = −0.31 ± 0.05 Gy (1 standard deviation) and *b* = 2.5 ± 0.1 Gy, for LET given in keV *μ*m^−1^ and ${z}_{F,\max }$—in Gy. These data are shown in figure 1.

*Photons*. From a review on photon relative biological effectiveness (Vassiliev *et al* 2020), we took a dataset comprising 139 measured (*α*, *β*) pairs for photon energies from 0.27 keV to 1.25 MeV and average delta-electron LET values from 0.40 to 10.5 keV *μ*m^−1^. The linear regression coefficients in this case were: *a* = −0.37 ± 0.12 Gy and *b* = 2.4 ± 0.1 Gy. These data are also shown in figure 1. The photon regression was remarkably close to the proton regression.

*Heavy ions*. Our analysis for heavy ions was based on the data reported by Furusawa *et al* (2000). It included cell survival data for HSG, V79 and T1 cells irradiated with ^3^He, ^12^C and ^20^Ne ions, in aerobic and hypoxic conditions. In our dataset, we included only aerobic data. We separated all the data into two subsets: ^3^He and heavier ions. The helium subset comprised 20 (*α*, *β*) pairs for V79 cells and 9 pairs for HSG cells. LET values ranged from 18.5 to 90.8 keV *μ*m^−1^. We fit all these ion data with second-order polynomials. For V79 cells the result was ${z}_{F,\max }=0.061\cdot {\left(\mathrm{ln}{\mathrm{LET}}\right)}^{2}-1.4\cdot \mathrm{ln}{\mathrm{LET}}+5.4$, where LET is in keV *μ*m^−1^ and ${z}_{F,\max }$ is in Gy. For HSG cells the best-fit polynomial was ${z}_{F,\max }=0.32\cdot {\left(\mathrm{ln}{\mathrm{LET}}\right)}^{2}-2.8\cdot \mathrm{ln}{\mathrm{LET}}+6.4$. These data are shown in figure 1.

**Figure 2.**
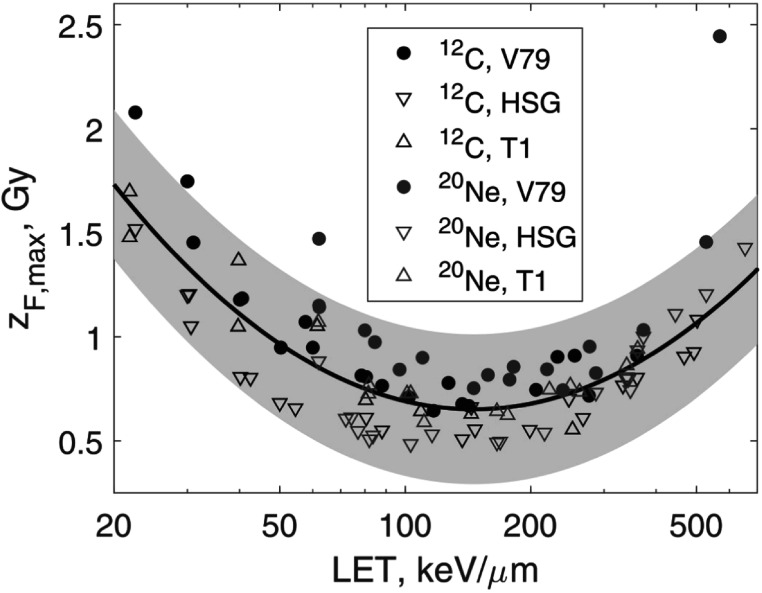
Upper bounds for *z*_*F*_ derived by method 1 (equation (16)) from experimental *α* and *β* values (Furusawa *et al* 2000). Circles and triangles show data for ^12^C and ^20^Ne ions for V79, HSG and T1 cells. The solid line shows a fit of data by a second-order polynomial. The shaded area is the 95% confidence interval of the fit.

The heavy ion subset comprised 82 measured (*α*, *β*) pairs for V79, HSG and T1 cells irradiated with ^12^C and ^20^Ne ions. The LET range was 21.8–654 keV *μ*m^−1^. The data are shown in figure 2. In this case: ${z}_{F,\max }=0.27\cdot {\left(\mathrm{ln}{\mathrm{LET}}\right)}^{2}-2.7\cdot \mathrm{ln}{\mathrm{LET}}+7.4$. The data in figure 2 are overall consistent with our results shown in figure 1. However, the trend seen in figure 1 is reversed for LET values exceeding 200 keV *μ*m^−1^. This is related to the well-known properties of *α* as a function of LET.

#### 3.1.2. Method 2

**Figure 3.**
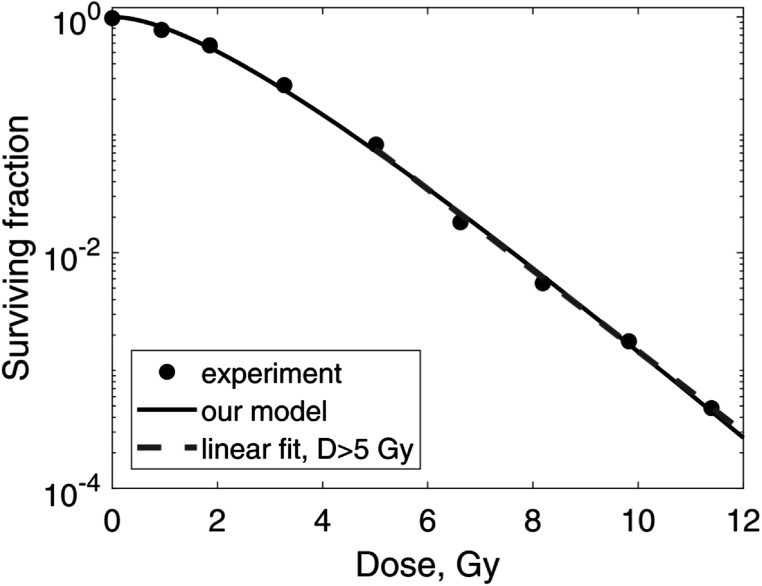
Survival of V79 cells irradiated with 55 kV x-rays. Fit of experimental data (Elkind and Sutton 1959, circles) with our model, equation (4) (solid line), is shown. The dashed line shows a fit of the experimental data above 5 Gy with a linear model.

*55* *kV *x-rays. The measured cell survival data (Elkind and Sutton 1959) are shown in figure 3. We fit these data with the three-parameter version of our model (equation (4)), in which we set *s*_*k*_ = 0 for all *k* > 2 and the fitting parameters were *s*_1_,*s*_2_, and *z*_*F*_. The best fit values were *s*_1_ = 1.0 ± 10^−6^, *s*_2_ = 0.35 ± 0.16, *z*_*F*_ = 1.0 ± 0.1 Gy. This fit is shown in figure 3 with a solid line. We then added one more paremeter, *s*_3_, and repeated the fit. The best-fit value for *s*_3_ was 3.3 · 10^−5^ ± 0.09. We concluded that *s*_3_ can be set to zero, and chose the three-parameter fit as the best option for these data. In this example it takes two or three hits to kill a cell. The average LET of delta-electrons produced by x-rays of this energy was 2.29 keV *μ*m^−1^ (Vassiliev *et al* 2020). Then, using the linear regression shown in figure 1, we found that ${z}_{F,\max }$ was 2.0 ± 1.3 Gy. Hence, *z*_*F*_ for 55 kV x-rays that we found directly by fitting the measured cell survival data was within the bounds given by equation (16).

**Figure 4.**
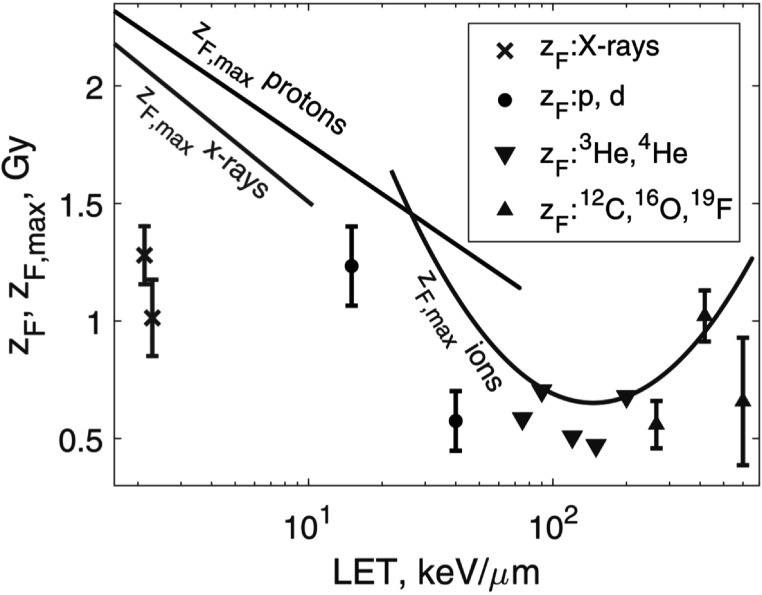
Comparison of the upper bound ${z}_{F,\max }$ calculated by method 1 (equation (16)) with *z*_*F*_ derived by method 2. The symbols show method 2 *z*_*F*_ data for several particle types, as indicated in the legend. One x-ray point (*z*_*F*_ = 1.0 Gy) was derived from Elkind and Sutton (1959), all the other points are from Miller *et al* (1995). The solid lines are regression models for ${z}_{F,\max }$ shown in figures 1 and 2. The red line indicates x-rays; black line, protons and deuterium; blue curve, heavy ions. Error bars for helium ions are not shown because they are approximately the size of the symbols.

*Heavy ions*. Miller *et al* (1995) reported cell survival tables for protons, deuterium, ^3^He, ^4^He, ^12^C, ^16^O, and ^19^F ions with LET values from 3.8 to 600 keV *μ*m^−1^. That study included data for 250 kVp x-rays that we also included in our analysis. For each cell survival curve we identified an approximately linear segment and fit it with a linear model. From the slope of the line we found *z*_*F*_ following method 2. In figure 4, we compare these *z*_*F*_ values with the upper bounds ${z}_{F,\max }$ that we calculated by method 1. Figure 4 shows that our results for ${z}_{F,\max }$ (method 1) are consistent with *z*_*F*_ values derived by method 2. Figure 4 provides an order-of-magnitude estimate of *z*_*F*_ for various particle types: 1 Gy. This value means that in most cases, one hit causes significant damage. We therefore consider each hit as an event causing damage, lethal or sublethal.

### 3.2. Calculation of SV size

*Photons*. For *y*_*F*_, we used measured data (Kliauga and Dvorak 1978) for monoenergetic photons from 12 keV to 1.25 MeV, and spherical volumes with radii from 0.12 to 3.85 *μ*m. Using this *y*_*F*_ table we found *R*_min_ for three photon energies: 12 keV, ${R}_{\min }=0.29$ *μ*m; 140 keV, ${R}_{\min }=0.18$ *μ*m; 1.25 MeV ^60^Co, ${R}_{\min }=0.11$ *μ*m. The confidence intervals could not be calculated because they extended too far outside the range of the *y*_*F*_ table. These results are consistent with a study on x-ray relative biological effectiveness (Vassiliev *et al* 2020) that estimated by a different method the SV diameter to be on the order of 0.1–1 *μ*m.

**Figure 5.**
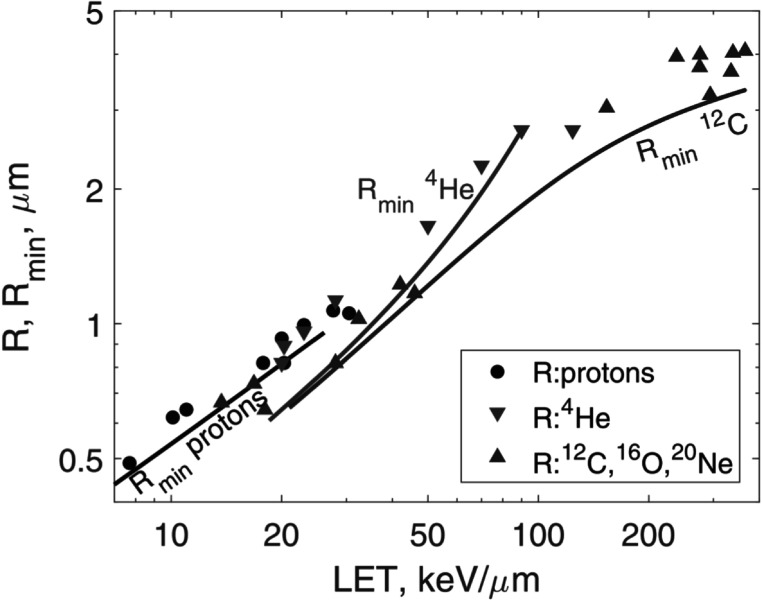
Comparison of the lower bound for SV radius, *R*_min_ derived from equation (17), with radii *R* derived from experimental inactivation cross-sections for V79 cells (Belloni *et al* 2002). The symbols show *R* for several particle types as indicated in the legend. The solid lines are *R*_min_ values calculated using equation (17) and regression models for ${z}_{F,\max }$ shown in figures 1 and 2. The black line indicates protons; red line, ^4^He; blue line, ^12^C.

*Protons*. First, we calculated *R*_min_ for 100 MeV protons. We used tables of basic microdosimetric quantities (Vassiliev *et al* 2019) calculated with Monte Carlo. Taking *y*_*F*_ from these tables, we found that ${R}_{\min }=0.12$ *μ*m (95% CI 0.099–0.15 *μ*m). For protons with higher LET values, *R*_min_ were higher. We compared this increase with a similar trend observed for the inactivation cross section, *σ*. For this comparison, we used *σ* for V79 cells summarized by Belloni *et al* (2002) and compared $R=\sqrt{\sigma /\pi }$ with our *R*_min_. For volumes larger than those included in the Monte Carlo tables, we used the approximation *y*_*F*_ ≈ LET (Vassiliev *et al* 2019). The calculated *R*_min_ and *R* values derived from experimental *σ* are shown in figure 5. Our data closely followed the trend exhibited by the experimental data.

*Heavy ions*. For ^4^He and ^12^C ions we used *y*_*F*_ calculated with Monte Carlo (Nikjoo *et al* 2016). These data are limited to a spherical SV of 0.5 *μ*m radius. However, *y*_*F*_ as a function of SV size is known to plateau for radii exceeding a few tenths of a micrometer. For example, for protons, when the radius increases from 0.25 to 0.5 *μ*m, *y*_*F*_ decreases only by 3% (Vassiliev *et al* 2019). Hence, we neglected variation of *y*_*F*_ with the SV size. Our data for heavy ions shown in figure 5 were consistent with the inactivation cross-sections (Belloni *et al* 2002).

Given that SV size increases with increasing LET (figure 5), SV should not solely be interpreted as the size of a subcellular structure. SV also accounts for the spatial extent of damage and the range of interaction between damaged sites. Measurements of *γ*H2AX foci sizes (Nakajima *et al* 2013) support this conjecture. For 200 kVp x-rays, the median foci width was 0.45 *μ*m (range 0.3–1.0 *μ*m). For foci formed within tracks of 200 keV *μ*m^−1^ iron ions, the median width was 1.8 *μ*m (range 1.6–2.9 *μ*m).

### 3.3. Accumulation of SRD

One parameter, *D*/*z*_*F*_, determines the number of cells receiving a given number of hits (equations (2), (3)). Cell survival data for V79 cells irradiated with 55 kV x-rays (Elkind and Sutton 1959) were well suited for calculating *z*_*F*_ with method 2 because these data have a well-defined linear segment (figure 3). Owing to this property, we obtained a reliable estimate: *z*_*F*_ = 1.0 Gy. Simple calculations showed that in this case, at 1 Gy 37% of all cells remained undamaged and at 5 Gy this number was reduced to 0.7%.

Distribution of the number of hits only for surviving cells represents the extent of SRD. Our model predicted that the fraction of surviving cells that received zero hits decreases with dose:

\begin{eqnarray*}{f}_{0}=\displaystyle \frac{{h}_{0}}{S}={\left[1+{{Ns}}_{1}+\displaystyle \frac{{N}^{2}}{2}{s}_{2}+\displaystyle \frac{{N}^{3}}{3!}{s}_{3}+\ldots \right]}^{-1}.\end{eqnarray*}

The probability of a cell receiving *k* hits and surviving is *s*_*k*_ *h*_*k*_. Therefore, the fraction of surviving cells with *k* hits is

\begin{eqnarray*}{f}_{k}=\displaystyle \frac{{s}_{k}{h}_{k}}{S}.\end{eqnarray*}

**Figure 6.**
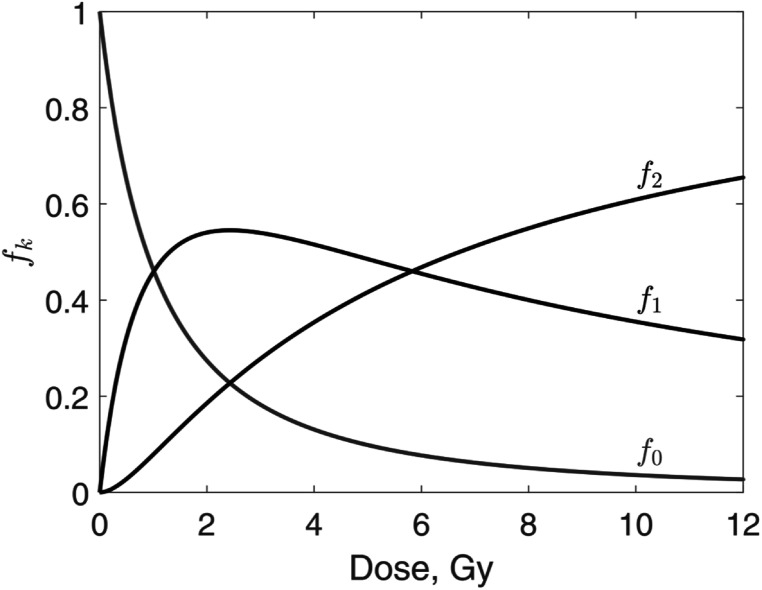
Fraction of surviving cells that received *k* hits, *f*_*k*_, *k* = 0, 1, 2. Data are shown for V79 cells irradiated with 55 kV x-rays (Elkind and Sutton 1959).

We calculated these quantities for 55 kV x-rays (Elkind and Sutton 1959). This case was considered in section 3.1.2 and the data are shown in figure 3. The results for *k* = 0, 1, 2 are shown in figure 6. For *k* > 2 we determined by the fit that *f*_*k*_ = 0. Starting at doses below 1 Gy, the initially homogeneous population of cells partitions into three subsets: undamaged cells, cells with one hit and two hits. These subsets have differing radiosensitivity: the undamaged cells can withstand up to two hits, whereas the subset that has received two hits, does not survive one additional hit. The relative size of this most radiosensitive subset increases with increasing dose leading to prevalence of the single-hit mechanism of cell kill. This leads to a constant slope of the survival curve, $\mathrm{ln}S$ versus dose.

Accumulation of SRD makes a cell population heterogeneous in terms of radiosensitivity. This heterogeneity should be accounted for in cell survival models. Such models need to predict partitioning of a cell population into subsets based on levels of damage sustained by individual cells, and have a different survival probability for each of the subsets.

In two situations, some aspects of our analysis are not applicable: (1) damage repair significantly changes the distributions shown in figure 6; and (2) differences in radiosensitivity of cells receiving different numbers of hits are insignificant. We show below that these situations are uncommon. Furthermore, our formalism can be extended to explicitly account for repair. In the current version, the probabilities *s*_1_, *s*_2_, … account for repair and other factors affecting survival.

(1) Damage repair is slow compared with the time needed to deliver a dose fraction in radiotherapy: ‘the half-time sublethal damage repair in mammalian cells is about 1 h’ (Hall and Giaccia 2011). More recent studies (Foray *et al* 2005, Matsumoto *et al* 2019) reported half-times from a few minutes to over 2 h. To estimate the impact of SRD repair, we calculated the probability of a cell completing damage repair in the time interval Δ*t* between two consecutive hits. If the fraction delivery time is *t*_*f*_ and the dose rate is $\dot{D}$, then the average number of hits *N*_*f*_ during *t*_*f*_ is

\begin{eqnarray*}{N}_{f}=\dot{D}{t}_{f}{/z}_{F},\end{eqnarray*}

and the average time between hits is $\overline{{\mathrm{\Delta }}t}={t}_{f}{/N}_{f}$. Particle interaction with an SV is a Poisson process. Therefore, Δ*t* is exponentially distributed:

\begin{eqnarray*}f({\mathrm{\Delta }}t)=a\cdot {{\mathrm{e}}}^{-a{\mathrm{\Delta }}t},\end{eqnarray*}

where *f* is the probability density function for the time interval between two consecutive hits, and $a={\left(\overline{{\mathrm{\Delta }}t}\right)}^{-1}$. We assume exponential repair kinetics, with the repair rate constant $\mu =\mathrm{ln}2/$(repair half-time). This leads to the following formula for the probability of completing repair in the time interval between two hits:

\begin{eqnarray*}{P}_{R}=a{\int }_{0}^{{t}_{f}}{{\mathrm{e}}}^{-a{\mathrm{\Delta }}t}(1-{{\mathrm{e}}}^{-\mu {\mathrm{\Delta }}t}){\mathrm{d}}({\mathrm{\Delta }}t)=\displaystyle \frac{\mu }{\mu +a}-{{\mathrm{e}}}^{-{{at}}_{f}}\left(1-\displaystyle \frac{a{{\mathrm{e}}}^{-\mu {t}_{f}}}{a+\mu }\right).\end{eqnarray*}

Equation (22) is based on the total probability law (Ash 2008). The integrand is the product of probability *f*(Δ*t*) and conditional probability (the expresson in parentheses) of completing repair at any time between 0 and Δ*t*.

If a beam of 100 MeV protons delivers a 2 Gy fraction at a dose rate of 0.05 Gy s^−1^, and the repair half-time is 1 h, this probability is negligible: *P*_*R*_ = 0.0018. Hence, usually repair has little impact on SRD buildup in a cell during a fraction delivery.

(2) We can estimate the probabilities *s*_1_ and *s*_2_ of a cell surviving one and two hits from experimental *α* and *β*. To do so, we invert equations (7) and (10):

\begin{eqnarray*}{s}_{1}=1-\alpha {z}_{F}.\end{eqnarray*}

\begin{eqnarray*}{s}_{2}={s}_{1}^{2}-2\beta {z}_{F}^{2}.\end{eqnarray*}

To calculate *s*_1_ and *s*_2_ for photons and protons we used the same experimental sets of (*α*, *β*) pairs as in figure 1. For a given *z*_*F*_, equations (23) and (24) produced sets of (*s*_1_, *s*_2_) values. For *z*_*F*_ = 1 Gy, the median values were *s*_1_ = 0.79, *s*_2_ = 0.57 for photons, and *s*_1_ = 0.57, *s*_2_ = 0.26 for protons. For heavy ions, we used the same (*α*, *β*) sets as in figure 2 but limited to LET values of 100–200 keV *μ*m^−1^. For heavy ions *z*_*F*_ tends to be lower (figure 4), hence we calculated *s*_1_ and *s*_2_ for *z*_*F*_ = 0.5 Gy. In this case, the probabilities were *s*_1_ = 0.46, *s*_2_ = 0.18 for the combined ^12^C and ^20^Ne data. These results showed that each hit substantially reduces the probability of cell survival.

## 4. Conclusions

We proposed a novel cell survival model. It correctly predicts the basic properties of cell survival curves. It is consistent with the LQ model at low doses, and at high doses it predicts the transition to a constant slope. Formally, our model has an infinite number of parameters: *s*_1_, *s*_2_,…,*z*_*F*_. However, our review of experimental data showed that even for photons, *s*_*k*_ can in some cases be set to zero for all *k* > 2, and that the number of parameters decreases with increasing LET. Therefore, our model can be used as an alternative to the LQ formula for curve fitting, especially for data that extend to low survival levels.

We used the model to investigate accumulation of SRD and its impact on cell survival. We found that SRD is an important factor affecting cell survival even at low doses of about 1 Gy. It becomes a crucial factor at doses above a few Grays. Cells receiving different amounts of SRD have significantly different radiosensitivity and, because cells are affected at random, accumulation of SRD makes cell populations heterogeneous in terms of radiosensitivity. Cell survival models should account for this heterogeneity. We have also estimated the SV size. Our data were consistent with experimental inactivation cross-sections in suggesting that SV size increases with increasing LET. This is different from the standard microdosimetric approach, in which the SV size is a constant (Rossi and Zaider 1996, Kase *et al* 2006). Our *R*_min_ varied from 0.1 *μ*m for ^60^Co gamma rays to 3 *μ*m for 300 keV *μ*m^−1^ ^12^C ions. Finally, we provided evidence supporting a simple mechanism for the transition of cell survival curves to constant-slope lines at high doses: accumulation of SRD increases cell radiosensitivity and above a certain dose repair is inhibited in most cells, which results in transition to a constant slope.

Our model is primarily intended for hadron therapy treatment planning. It advances RBE calculations beyond the LQ model and offers a formalism that has a potential for improving RBE predictions at high dose levels used in hypofractionated treatments. For clinical implementation of our model it is necessary to validate it and determine its parameters for different particles and x-rays or gamma radiation as a reference radiation. This should cover clinically relevant LET and dose ranges, and tissue types.
